# Inhibition of Fibroblast Activation in Uterine Leiomyoma by Components of *Rhizoma Curcumae* and *Rhizoma Sparganii*

**DOI:** 10.3389/fpubh.2021.650022

**Published:** 2021-03-01

**Authors:** Yewen Feng, Yumin Zhao, Yao Li, Teng Peng, Yu Kuang, Xingming Shi, Gang Wang, Fu Peng, Chenghao Yu

**Affiliations:** ^1^Basic Medicine College, Chengdu University of Traditional Chinese Medicine, Chengdu, China; ^2^Department of Pediatrics, The Second Hospital Affiliated Shaanxi University of Chinese Medicine, Shaanxi, China; ^3^Suining Traditional Chinese Medicine Hospital, Sichuan, China; ^4^Medical College of Georgia, Augusta University, Augusta, GA, United States; ^5^National Engineering Research Center for Biomaterials, Sichuan University, Sichuan, China; ^6^West China School of Pharmacy, Sichuan University, Sichuan, China; ^7^State Key Laboratory of Southwestern Chinese Medicine Resources, Sichuan, China

**Keywords:** fibroblast activation protein, *Rhizoma Curcumae*, *Rhizoma Sparganii*, tumor-associated fibroblasts, traditional Chinese medicine, uterine leiomyoma

## Abstract

**Background:** The herbs *Rhizoma Curcumae* and *Rhizoma Sparganii* (RCRS) are often used in traditional Chinese medicine for the treatment of uterine leiomyoma (UL). The effectiveness of RCRS for the treatment of UL has been confirmed in our previous studies.

**Purpose:** This study aimed to investigate the molecular mechanism by which RCRS inhibits the activation of fibroblast activation protein (FAP) and prevents UL in rats.

**Study Design and Methods:** A Sprague Dawley (SD) rat model of UL was established via estrogen and progesterone load combined with external stimulation. Histological analyses, enzyme-linked immunosorbent assays, and western blotting were performed to evaluate the effect of RCRS on UL and elucidate its mechanism of action.

**Results:** Our data showed that the treatment of SD rats with RCRS significantly reduced the expression of extracellular matrix component collagen, FAP, and transforming growth factor beta (a FAP-activating factor) and the phosphorylation of the cell proliferation pathway-related signaling factors AKT/MEK/ERK.

**Conclusion:** Our results suggest that RCRS is effective in the prevention and treatment of UL in rats, and RCRS may exert its functions by inhibiting the activation of tumor-associated fibroblasts and cell proliferation and by improving the tumor extracellular matrix.

## Introduction

Uterine leiomyoma (UL) is the most common type of benign reproductive tract tumor in women of childbearing age, with an incidence rate of ~70%, of which 25–50% of patients exhibit clinical symptoms (1). Because fibroblasts are the main stromal cells of UL, UL is also referred to as uterine fibroids (2). The principal drugs used in the clinical treatment of UL include mifepristone, letrozole, and vitamin D. Surgical treatment is currently the primary means of clinical treatment of UL, and hysterectomy is the only definitive cure for UL. However, these treatments have serious side effects; thus, it is important to identify effective and safe methods to treat UL.

*Rhizoma Curcumae* (RC) and *Rhizoma Sparganii* (RS) (RCRS) are frequently used in patients with gynecological diseases such as uterine fibroids, dysmenorrhea, and ovarian cysts to improve blood circulation and remove stasis. A combination of RC (*Curcuma phaeocaulis Valeton*) and RS (*Sparganium stoloniferum* Buch. -Ham.) is a representative medication for UL treatment in traditional Chinese medicine (TCM) (3). Chemical analyses have shown that RS mainly contains volatile oil, flavonoids, saponins, organic acids, and other functional ingredients (4). Total flavonoids are one of the major active ingredients of RS and possess pharmacological effects such as anticoagulation, antithrombotic, and anticancer effects. Zedoary turmeric oil is a volatile oil extracted from RC. As an effective antitumor drug, the main active ingredients of zedoary turmeric oil are curcumin, curcumin diketone, and β-elemene (5, 6).

RCRS, a combination of total flavonoids and zedoary turmeric oil, has a significant therapeutic effect on experimental UL (7). Our previous studies have confirmed that the therapeutic effects of RCRS on UL include improvement of pathological status, reduction of volume, and inhibition of myoma cell proliferation (7). Another study showed that the therapeutic effect of RCRS on UL might be related to the regulation of endocrine levels (8). The results of a metabolomics study confirmed that RCRS can reverse abnormal metabolism in rats. However, its mechanism of action remains poorly studied (9). Because of the multi-channel and multi-target characteristics of TCM, the key targets of RCRS are unclear and require investigation. ULs are composed of leiomyoma cells, fibroblasts, and a large number of extracellular matrices (ECMs) (10). Studies have shown that RCRS inhibits the proliferation of leiomyoma cells, but the mechanism remains to be elucidated. Most studies on UL have focused on leiomyoma cells, but the mechanisms underlying the activation of tumor-associated fibroblasts (TAFs) and UL development are unclear.

In this study, we used a rat model of qi stagnation and blood stasis UL, in which UL was induced via estrogen and progesterone load combined with external stimulation. The way of modeling has been verified to be reliable and repeatable (11). This model is more suitable for TCM experiments according to the basic theory of TCM combined with western medical theory and experimental zoology. Because fibroblast activation leads to the development of UL, we assumed that RCRS inhibits UL by inhibiting TAF activation. The results of this study should provide an experimental basis for using TCM for the treatment of UL.

## Materials and Methods

### Preparation of Herbal Extract

The volatile oil of RC was prepared and validated as described in our previous study (11). A voucher specimen (NO.EZ 0704) was deposited at the Chengdu University of Traditional Chinese Medicine, Chengdu, China. RS was identified by Professor Teng Peng of the Department of Pharmacy, Chengdu University of Traditional Chinese Medicine. For preparation of total flavonoids of RS, we weighed RS powder, added eight volumes of 70% ethanol, heated and extracted twice (1.5 h each time), recovered the extract after filtration. Concentrate the extract with a rotary evaporator and put it in a water bath for drying. After dissolving and diluting the extract with 70% ethanol, it was poured into a macroporous resin for filtration, and the filtrate was collected and dried the extract in a 45°C drying oven to a constant weight. A voucher specimen (NO.SL 0704) was deposited at the Chengdu University of Traditional Chinese Medicine.

Three concentrations of the compound were used: 66.7% extract (6.67 g/kg), 33.3% extract (3.33 g/kg), and 16.7% extract (1.67 g/kg). All extracts were stored at 4°C until use.

### Preparation of Herbal Decoction

The herbs (RS:RC = 1:1) were crushed and soaked in water for 2 h. Herb fragments were decocted twice: for 1 h for the first extraction and 0.5 h for the second extraction. Following mixing and filtering, the decoction was concentrated to 0.67 g of crude drug/mg and stored at 4°C.

### Rats

Rat care and experimental procedures were performed under a protocol approved by the Animal Ethical Committee at the Chengdu University of Traditional Chinese Medicine (Animal Ethics Approval Number NO. 2017-08). Female specific pathogen-free Sprague Dawley (SD) rats [Animal License No. SYXK (Chuang) 2014-049] were purchased from Chengdu Dashuo Experimental Animal Tech (China). All rats were adaptively fed with *ad libitum* access to standard rodent diet and water for 4 days. All animals were anesthetized with 2% pentobarbital sodium (0.25 ml/100 g) and sacrificed by cervical dislocation after 5 weeks.

### Study Design

Seventy-two SD rats were randomly divided into nine groups: control group, model group, RCRS-treated groups (66.7, 33.3, and 16.7%), RC-treated group, RS-treated group, decoction-treated group, and a positive control group. Gongliuxiao capsule is used as a positive medicine. The UL model was established following adaptive feeding. The UL model is established by the method that has been verified before (11). The control group rats received no treatment. The remaining eight groups were administered diethylstilbestrol (1.35 mg/kg) intragastrically every day and 1 mg progesterone intramuscularly three times a week for 5 weeks.

At the beginning of the fourth week, the eight experimental groups of rats were injected subcutaneously with epinephrine hydrochloride (0.5 mg/kg/d), and one external stimulus was applied 4 h after the injection: (1) reversal of day and night, (2) continuous stimulation with 60 db noise for 3 h, (3) continuous hanging upside-down for 10 min, and (4) water bath at 5–10°C for 4 min. This lasted for 2 weeks; each stimulus was applied ≥twice in 2 weeks.

**Figure 1:**
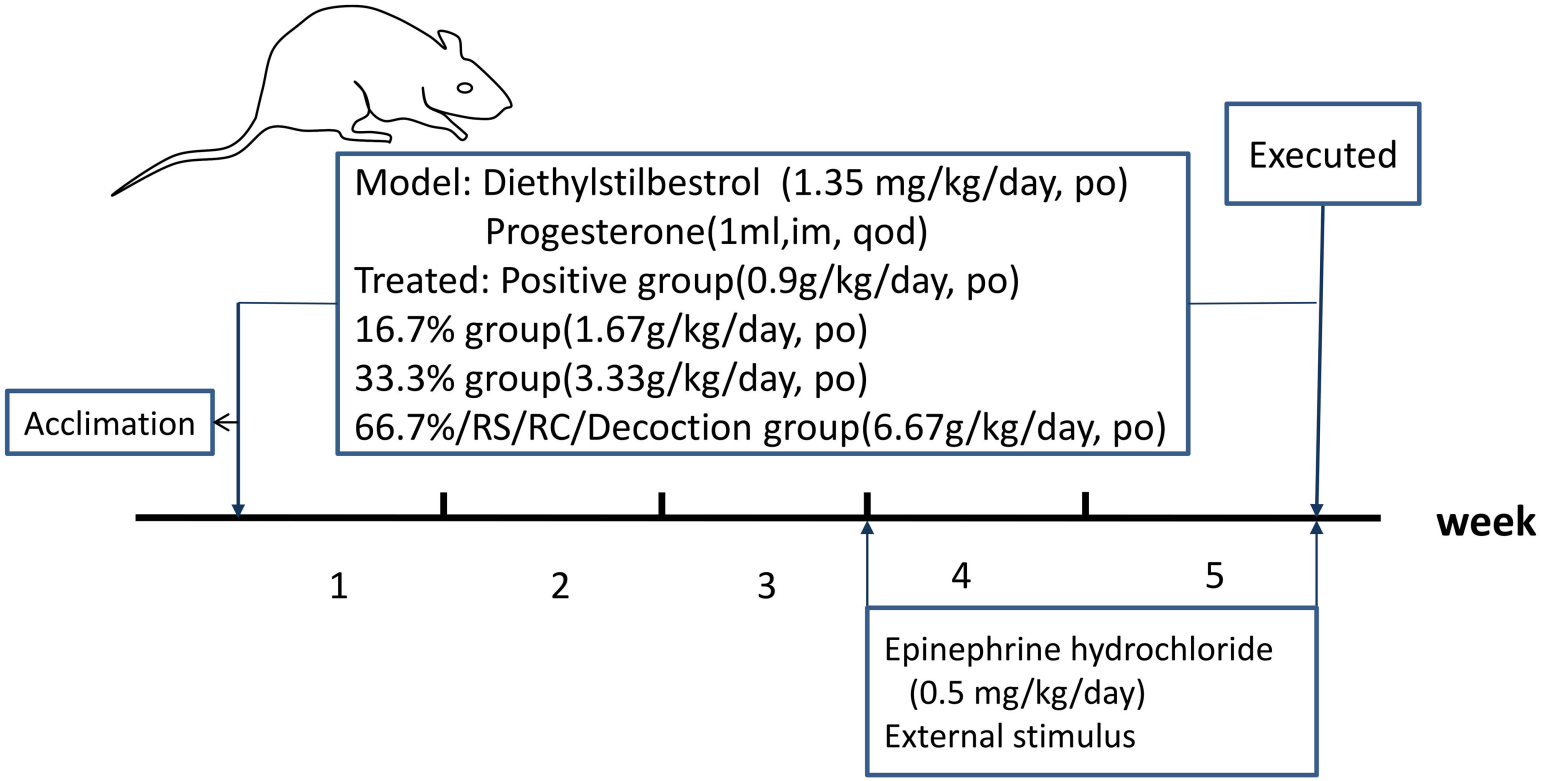
Schematic design of the experiment.

At the beginning of modeling, the drug was administered to the intervention groups at the same time, and the corresponding groups were administered separately (Figure 1). After 5 weeks, rat serum was collected for enzyme-linked immunosorbent assay (ELISA) after anesthesia; the rat uterus was divided into two parts: one for paraffin embedding and one for western blot analysis.

### Histological Analyses

For hematoxylin and eosin staining, the uterus was fixed overnight in 4% paraformaldehyde. Then, it was embedded in paraffin, sectioned, and stained.

For immunohistochemistry analysis, the paraffin blocks were sectioned and blocked with a blocking buffer. Immunostaining was performed using the primary antibodies estrogen receptor (ER; anti-rabbit, ab3206, Abcam), progesterone receptor (PR; anti-rabbit, ab16661, Abcam), and fibroblast activation protein (FAP; anti-rabbit, ab28244, Abcam) and visualized using goat anti-rabbit antibody (SP-9001, Beijing Zhong Shan Golden Bridge Biotechnology, China) and 3,3′-diaminobenzidine stain.

### Enzyme-Linked Immunosorbent Assays

Rat serum samples were collected, diluted, and divided into two aliquots. One aliquot was used to measure estradiol (E2) levels, and the other aliquot was used to measure progesterone (P) levels. The assays were performed using ELISA kits purchased from Nanjing Jiancheng Bioengineering Institute (#R20181226 and #R20181227 for E2 and P, respectively). The absorbance at 450 nm was measured using a Multiskan MK3 microplate reader (Thermo Fisher Scientific Inc., Waltham, MA USA).

### Western Blotting

Uterus tissues were cut, homogenized, and centrifuged, and the supernatant was aspirated for use. Protein determination was performed using a BCA protein quantification kit (PICPI23223, Thermo Fisher Scientific). Glyceraldehyde 3-phosphate dehydrogenase was used as a loading control for error correction.

### Statistical Analysis

Data were analyzed using one-way analysis of variance. The results are expressed as the mean + standard deviation. A *p*-value of <0.05 was considered statistically significant. A nonparametric test was used if the data did not conform to a normal distribution.

## Results

### Identification of Total Flavonoids

**Figure 2:**
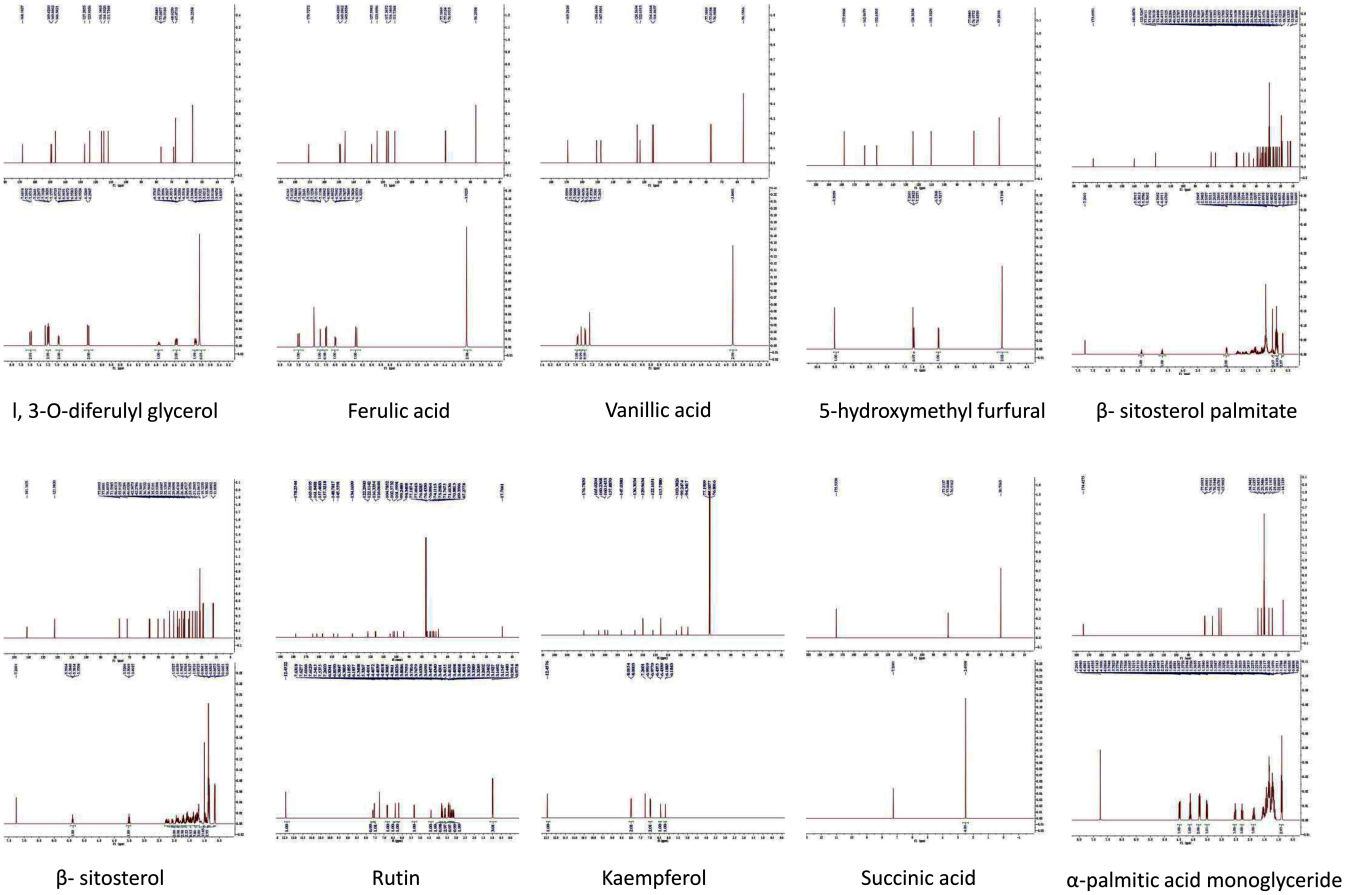
Compounds in the total flavonoids of Rhizoma Sparganii. Seventy percentage ethanol was used to extract the total flavonoids of Rhizoma Sparganii, and 10 compounds were identified, including 1,3-O-diferulyl glycerol, ferulic acid, vanillic acid, 5-hydroxymethyl furfural, β-sitosterol palmitate, β-sitosterol, rutin, Kaempferol, succinic acid and α-palmitic acid monoglyceride.

The yield of total flavonoids is 0.267%. A total of 10 flavonoid compounds were isolated from RS, and their structures were identified. These compounds included 1,3-O-diferulyl glycerol, ferulic acid, vanillic acid, 5-hydroxymethyl furfural, β-sitosterol palmitate, β-sitosterol, rutin, kaempferol, succinic acid, and α-palmitic acid monoglyceride (Figure 2).

### Establishment of the Rat Model

The rats in the model group displayed hair loss, decreased food intake, and slow increase in body weight.

**Figure 3:**
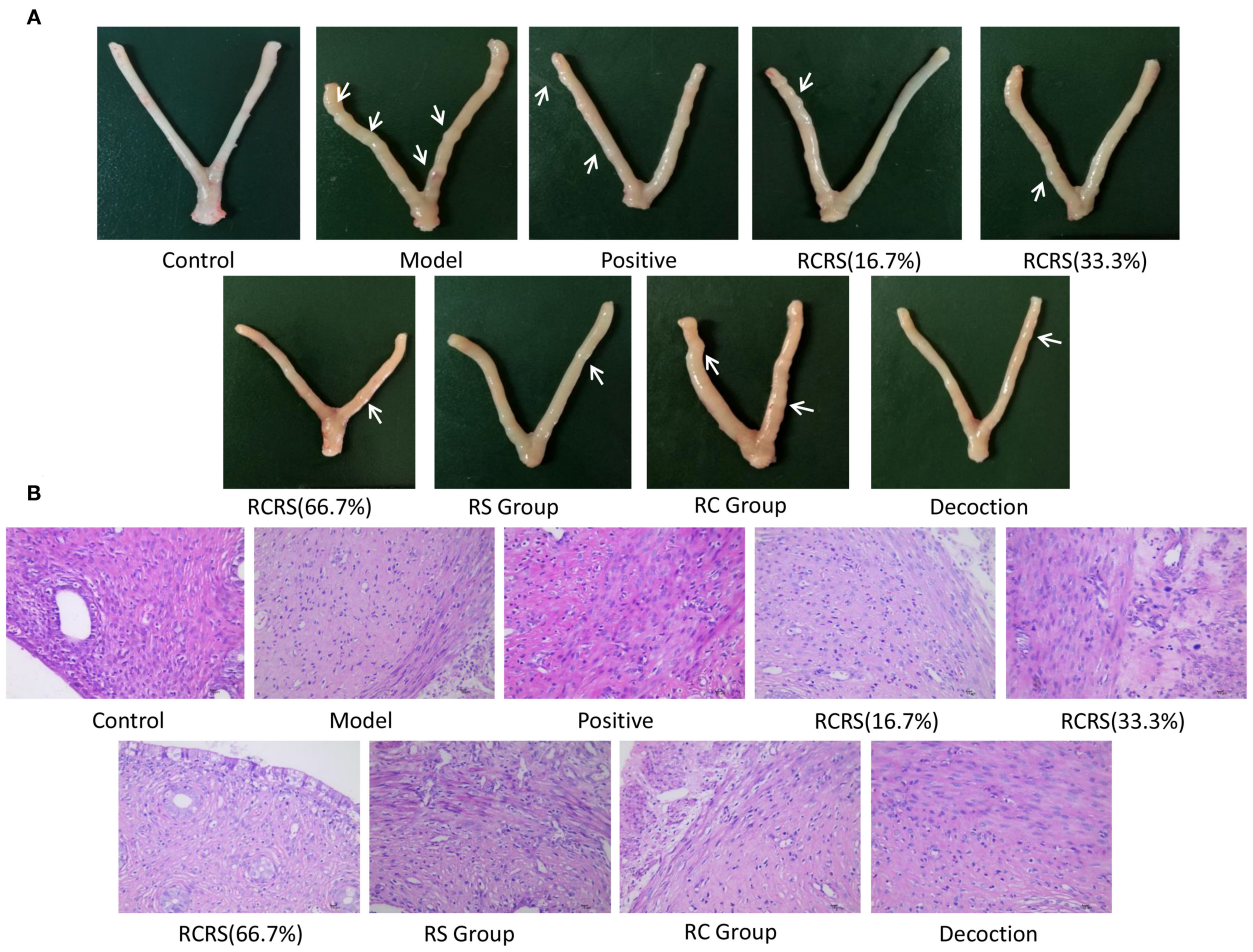
Preparation of uterine fibroid model by estrogen and progesterone load combining with external stimulation. **(A)** Representative uterus appearance of rats in each group. **(B)** Representative histological changes of uterus in each group (*n* = 8; magnification 400 ×).

The uterus of rats in the model group was dull, its texture was hard, and there were abnormalities, nodules, and macroscopic edema (Figure 3A). The transverse diameter and vertical diameter of the uterus in the model group increased, and the volume of the uterus also increased significantly.

**Figure 4:**
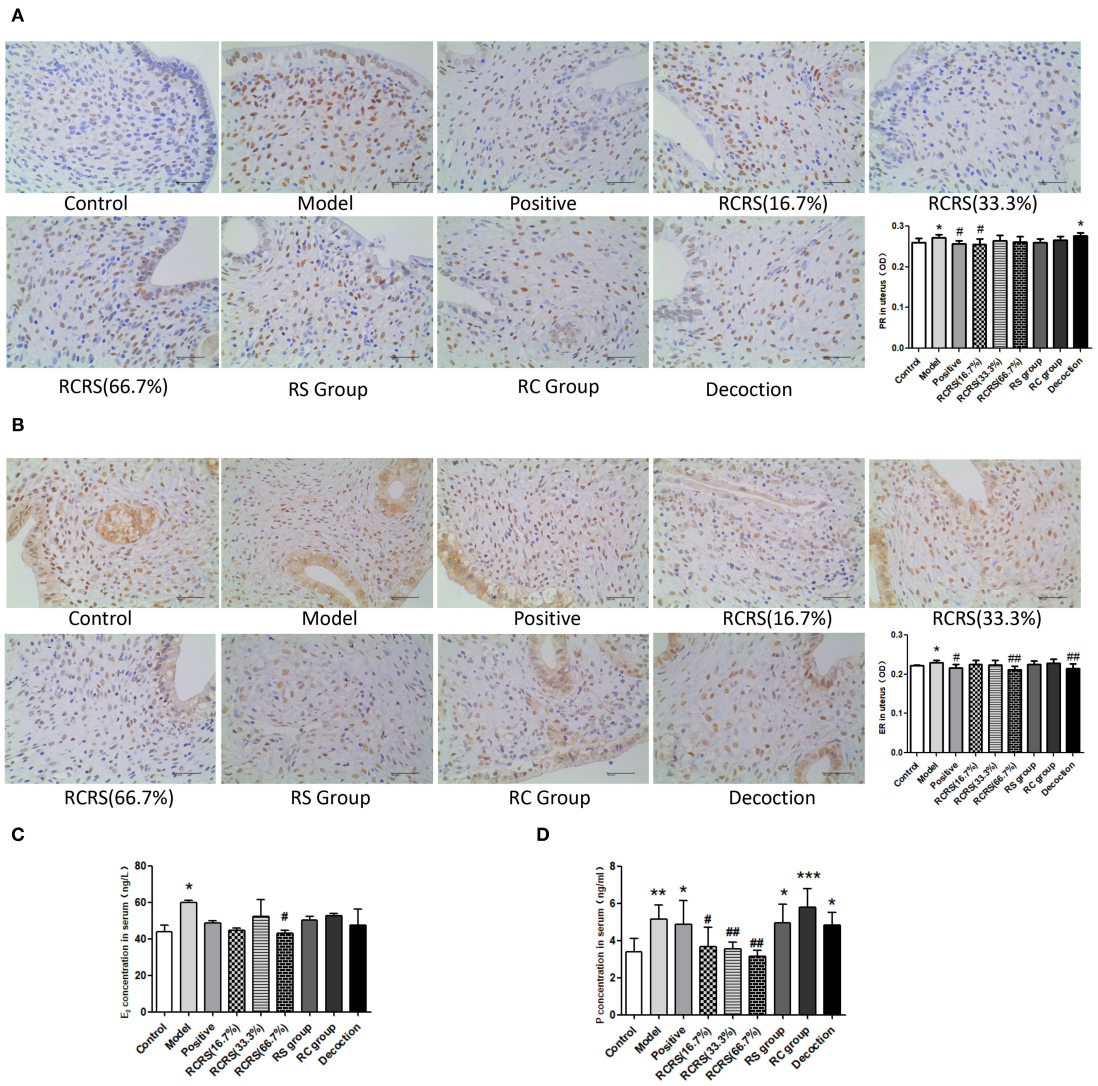
The result of estrogen and progesterone. Immunohistochemistry shows levels of **(A)** PR and **(B)** ER in uterus. The levels of **(C)** E_2_ and **(D)** *P* in serum were measured by ELISA (*n* = 8; mean + SD, ###*p* < 0.001, ##*p* < 0.01, #*p* < 0.5 compared with control group; ***p < 0.001, **p < 0.01, *p < 0.05 compared with control group; magnification 400 ×).

Pathological examination showed that compared with those in the control group, the uterine myometrial cells of the model group were disorganized, the thickness of the muscle layer was different, the outline of the muscle fibers was unclear, and the muscle fiber cells showed varying degrees of deformation and even necrosis (Figure 3B). This result is similar to the histopathological changes in uterine fibroids in human clinical patients. Meanwhile, the levels of estrogen and progesterone in the serum and uterus of the model group were significantly higher (Figures 4A–D).

These results demonstrated that the rat model of UL was successfully established.

### Effects of RCRS on the Appearance of the Uterus in Rats

Compared with the model group, the uterus of a rat that received medication was mostly symmetrical, the texture became soft, the thickness was uniform, and the uterine surface was smoother than the model group with no obvious swelling or ecchymoses. Several nodules can be seen on some uteri (Figure 3A).

### Effects of RCRS on Histological Changes of the Uterus

There were six (6/8) cases in the positive group, six (6/8) cases in the model group, six (6/8) cases in the RC-treated group, five (5/8) cases in the RS-treated group, three (3/8) cases in the (16.7%) RCRS-treated group, five (5/8) cases in the (33.3%) RCRS-treated group, and five (5/8) cases in the (66.7%) RCRS-treated group showing different degrees of degeneration (Figure 3B). The myometrium was disorderly arranged and varied in thickness. The muscle fibers were unclear and exhibited light-staining. The muscle fibers displayed varying degrees of degeneration and necrosis. The nucleus volume increased, and hyalinization could be seen in the muscle fibers. These results show that RCRS treatment can significantly improve the UL histological conditions.

### Effects of RCRS on the Expression of FAP in the Uterus

Immunohistochemistry analyses showed that the expression of FAP in the model group (*p* < 0.05) significantly increased compared with the control group. The expression of FAP in the RCRS-treated group (16.7%) was also increased. The effect of RCRS seemed to be dose-dependent as the low dose of the drug had a weaker inhibitory effect on FAP expression.

**Figure 5:**
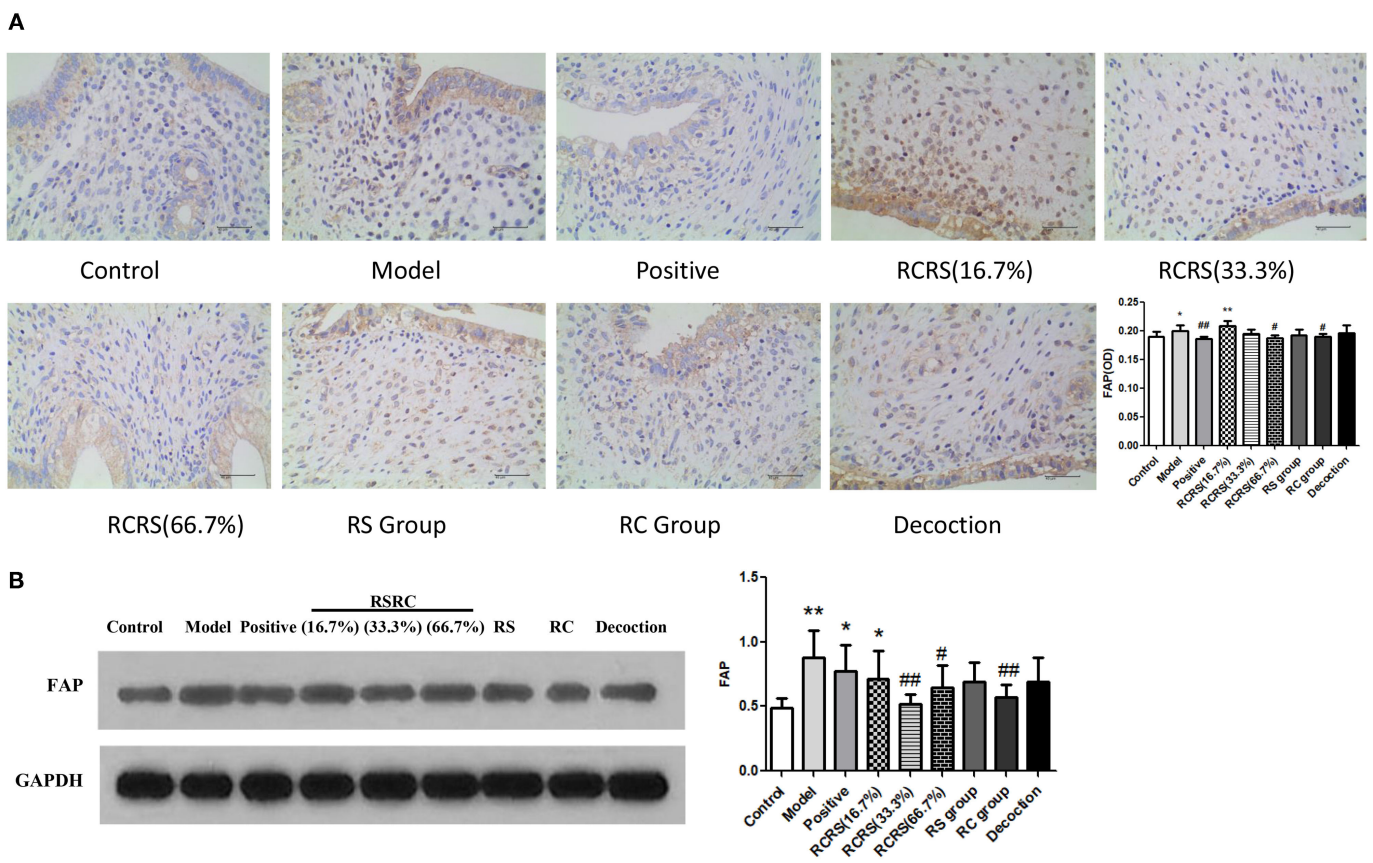
Effects of RCRS on the expression of FAP in uterus. **(A)** representative immunohistochemistry graphics of FAP in uterus. **(B)** representative western blotting band of FAP (*n* = 8; mean + SD, ##*p* < 0.01, #*p* < 0.5 compared with model group; **p < 0.01, *p < 0.05 compared with control group; magnification 400 ×).

Compared with the model group, FAP expression was significantly decreased following RCRS (66.7%) or RC treatment (*p* < 0.05). The positive control group showed a similar inhibitory effect on FAP expression (*p* < 0.01) (Figure 5A).

Western blot analysis results are similar to Immunohistochemistry results. FAP expression was significantly decreased following RCRS (33.3%) (66.7%) or RC treatment (*p* < 0.05), compared with the model group (Figure 5B).

Effects of RCRS on the expression of transforming growth factor beta (TGF-β) in the uterus.

Western blot analysis showed that the expression of TGF-β was significantly increased in the model group compared with the control group (*p* < 0.01).

**Figure 6:**
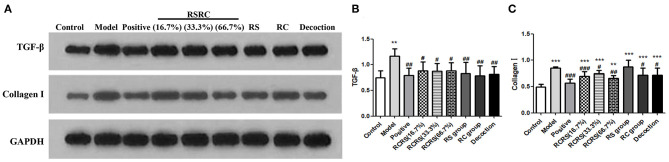
Effects of RCRS on the expression of TGF-β and extracellular matrix in uterus. **(A–C)** Representative western blotting band of TGF-β, collagen I (*n* = 6; mean + SD, ###*p* < 0.001, ##*p* < 0.01, #*p* < 0.5 compared with model group; ***p < 0.001, **p < 0.01 compared with control group).

Compared with the model group, the expression of TGF-β in the uterus in all drug intervention groups was significantly decreased (*p* < 0.05). However, no statistical differences were detected in the control group. In the positive group, the expression of TGF-β was similar to that of each drug intervention group (*p* < 0.01) (Figures 6A,B).

### Effects of RCRS on the Proliferation Signaling Pathway in the Uterus

**Figure 7:**
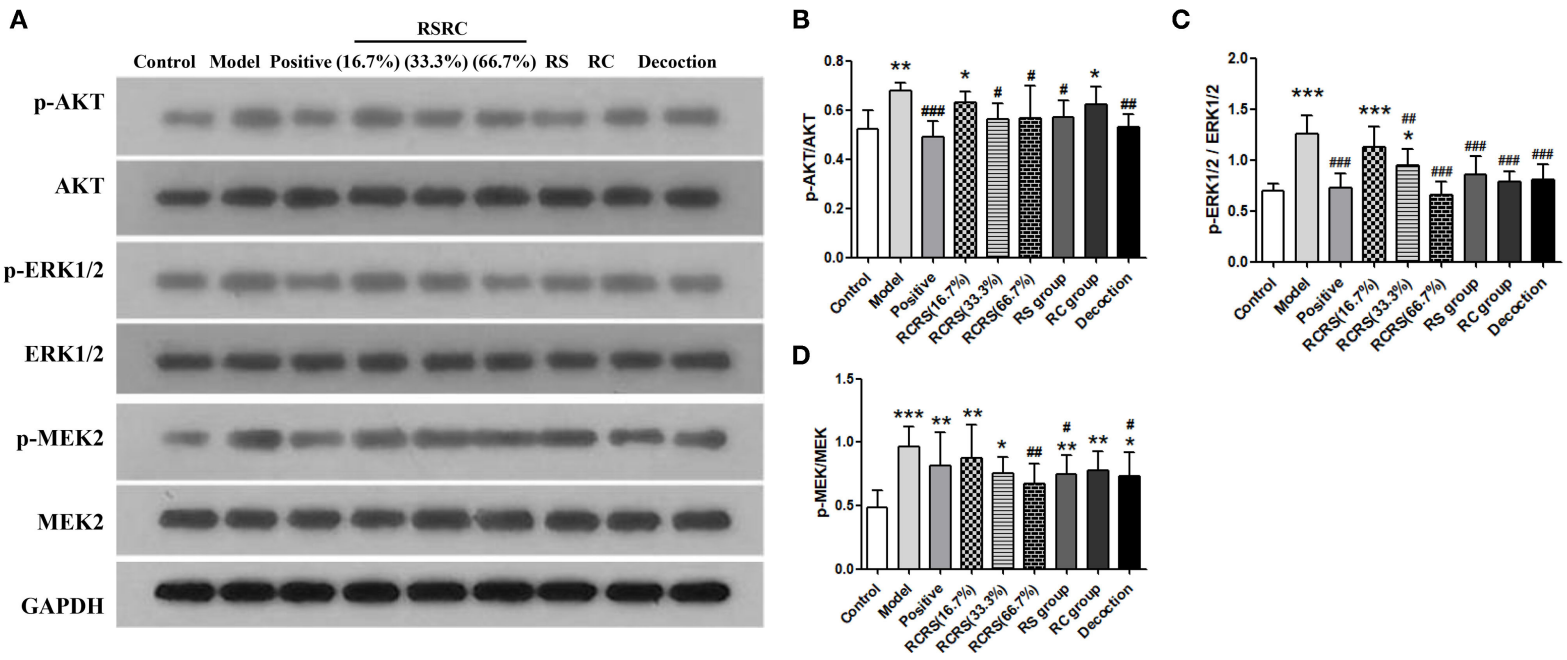
Effects of RCRS on the proliferation signaling pathway in uterus. **(A–D)** Representative western blotting band of p-AKT, AKT, p-ERK1/2, ERK1/2, p-MEK2 and MEK2 (*n* = 6; mean + SD, ###*p* < 0.001, ##*p* < 0.01, #*p* < 0.5 compared with model group; ***p < 0.001, **p < 0.01, *p < 0.05 compared with control group).

To investigate the effects of RCRS on the expression of proteins regulating cell proliferation and apoptosis, we analyzed the expression of AKT, ERK1/2, and MEK via western blot. The results showed that RCRS treatment had no significant effect on the expression levels of these proteins. However, compared with those in the control group, the phosphorylation levels of AKT, ERK1/2, and MEK in the model group were significantly increased (*p* < 0.001; *p* < 0.001). The phosphorylation levels of AKT, ERK1/2, and MEK were significantly reduced in the drug intervention group compared with those in the model group. The positive group had similar effects on AKT and ERK1/2 phosphorylation (*p* < 0.01; *p* < 0.001; *p* < 0.05) (Figures 7A–D).

### Effects of RCRS on the ECM in the Uterus

To study the effect of RCRS on the ECM of rat uterus, the expression of collagen I, an important component of the ECM, were analyzed by western blot. Compared with the control group, the expression of collagen I protein in the model group was significantly increased (*p* < 0.001; *p* < 0.05).

Compared with the model group, except for the RS-treated group, the drug intervention groups reversed the expression of collagen I to varying degrees (*p* < 0.05). This difference was particularly significant in the RCRS-treated (16.7%)/(66.7%) groups (*p* < 0.01). Compared with the model group, the level of collagen I in the positive group was also decreased (*p* < 0.001) (Figures 6A,C).

## Discussion

ULs, also known as fibroids, affect up to 70% of women before menopause and cause various clinical problems, such as excessive uterine bleeding, dysmenorrhea, infertility, and miscarriage (12). Uterine fibroids are considered estrogen-dependent tumors, and their development is closely related to the coordination of estrogen and progesterone. However, to the best of our knowledge, no report has evaluated the effects of RCRS on the TAFs of UL. Therefore, in this study, we aimed to address this issue using a well-established rat model of UL. Morphological and pathologic data showed that RCRS treatment significantly improved the symptoms of UL. Accordingly, we further examined whether RCRS improves UL by inhibiting TAF activation. As shown previously, approximately 80% of fibroblasts are activated in tumor tissues, and FAP plays a crucial role in fibroblast activation (13, 14). Santos et al. showed that the deletion of the *FAP* gene in TAF significantly decreased tumor cell proliferation activity, suggesting that FAP plays a key role in promoting tumor cell proliferation through TAF activation (15). Indeed, the western blotting results showed that the expression of FAP in the uterus was significantly decreased in the RCRS component compatibility, indicating that FAP can be inhibited by RCRS component compatibility, which inhibits the activation of UL fibroblasts.

TGF-β has pleiotropic effects and plays a key role in cell growth, differentiation, and immune response, and TGF-β1 has a significant influence on pathological changes of fibroids (16). TGF-β can promote TAF activation and induce normal fibroblast transformation (17). Our data showed that the expression level of TGF-β1 increased in the uterus in the model group, suggesting that increased FAP expression occurs due to the increase in TGF-β expression. RCRS treatment reversed these effects, suggesting that RSRC inhibits TGF-β expression and thus UL development.

Abnormal cell proliferation is one of the characteristics of uterine fibroid formation (18). The PI3K/AKT and MAPK/ERK signaling pathways are classical pathways related to cell proliferation and apoptosis (19, 20). It has been reported that AKT inhibitors can activate the AKT pathway in uterine fibroids, which increases the phosphorylation level of AKT compared with that in normal uterine tissue, suggesting that AKT is a potential biomarker for UL (21). Our results showed that the phosphorylation levels of AKT, ERK, and MEK were elevated in the model group, whereas those of AKT, ERK, and MEK were reduced after treatment with RCRS component compatibility. It has been shown that silencing of the TAF phenotype protein FAP can decrease the phosphorylation levels of AKT, ERK, and MEK (22), indicating that AKT, ERK, and MEK phosphorylation levels are associated with *FAP* expression and TAF activation; this result is consistent with our findings. These results indicate that the therapeutic effects of the total flavonoids of RS, volatile oil of RC, and their compatibility on UL may be related to the inhibition of FAP of TAF.

ULs are mainly composed of fibroid cells, fibroblasts, and ECM. ECM mainly includes collagen I, fibronectin, and laminin. A large amount of ECM deposition in uterine fibroid tissue is an important factor in the promotion of tumor development and metastasis (2). Tumor stroma is an important factor in promoting tumor development and metastasis. It is mainly composed of ECM, fibroblasts, inflammatory cells, and vascular smooth muscle cells.

Additionally, fibroblasts are in a static state in normal tissues and are stimulated by factors such as trauma to secrete a large amount of ECM protein (23–25). Therefore, the effects of RCRS component compatibility on collagen I in the uterine tissue of UL model rats were investigated. The results showed that the expression level of collagen I was reduced by the total flavonoids of RS and volatile oil of RC after treatment. However, the ability of the RCRS component compatibility to regulate the expression of collagen I in uterine tissue suggests that its mechanism of action for UL treatment may be related to RCRS component compatibility inhibiting TAF activation by inhibiting FAP expression.

RCRS are a commonly used drug pair in TCM. They are often used in 1:1 combination to treat various gynecological diseases. The compatibility of TCM can improve the performance of RCRS and enhance the therapeutic effect. Some scholars have separated and identified components in RCRS and have found that the mixed liquid contains more active ingredients than the simple RC/RS liquid and that the active ingredients extracted from the mixed liquid have better antioxidation and antitumor effects. In the experiment, a group of RCRS component compatibility was set up, and a single component group of total flavonoids of RS and a single component group of volatile RC oil were also set up. The results showed that the P level in the serum of UL rats in the RCRS component compatibility was significantly decreased and the PR, FAP, collagen I, and TGF-β levels in UL rat uterus tissues were significantly inhibited. Comprehensive analysis showed that the RCRS component compatibility was superior to the one component of the total flavonoids of RS and volatile oil in RC for the treatment of UL. Modern medicine uses a combination of drugs in the process of treating diseases, and the treatment effect is often better than that of a single drug. In TCM, the concept of drug combination has been practiced for thousands of years and has been validated in the clinical treatment of several diseases. Our experimental results also illustrate the scientific and effective theory of the compatibility of TCM.

## Conclusion

A rat UL model was established to evaluate the effect of RCRS on UL. The results show that RCRS can significantly improve the macroscopic and pathological state of the uterus in rats with UL, reduce the expression levels of TGF-β and FAP in the uterus, decrease the expression levels of signal factors (AKT, ERK, and MEK) in the cell proliferation-related pathway, and regulate the secretion of collagen I in the ECM. The findings that RCRS achieves the effect of treating UL by inhibiting the activation of TAF, the expression of factors in the cell proliferation pathway (AKT, ERK, and MEK), and collagen I in the ECM. The data may provide a new idea and therapeutic approach for UL and lay the foundation for further investigation for the treatment of UL.

## Data Availability Statement

The raw data supporting the conclusions of this article will be made available by the authors, without undue reservation.

## Ethics Statement

The animal study was reviewed and approved by the Animal Ethical Committee at the Chengdu University of Traditional Chinese Medicine (Animal Ethics Approval Number NO. 2017-08).

## Author Contributions

YF: validation, investigation, and writing-original draft. YZ: validation, data curation, and writing-original draft. YL: ideas and methodology. TP: resources. YK: resources. XS: writing- reviewing and editing. GW: writing- reviewing and editing. FP: conceptualization, writing-reviewing and editing. CY: conceptualization, writing- reviewing and editing, and project administration. All authors contributed to the article and approved the submitted version.

## Conflict of Interest

The authors declare that the research was conducted in the absence of any commercial or financial relationships that could be construed as a potential conflict of interest.

## Abbreviations

UL: uterine leiomyoma
TCM: traditional Chinese medicine
RCRS: *Rhizoma Curcumae* and *Rhizoma Sparganii*
ECM: extracellular matrix
TAF: tumor-associated fibroblasts
ER: estrogen receptor
PR: progesterone receptor
E2: estradiol
P: progesterone
FAP: fibroblast activation protein
TGF-β: transforming growth factor beta.
