# Supplementary figures and images for: Inhibition of Fibroblast Activation in Uterine Leiomyoma by Components of Rhizoma Curcumae and Rhizoma Sparganii
Source: Front Public Health. 2021 Mar 1;9:650022. doi: 10.3389/fpubh.2021.650022 (PMC7957009; doi:10.3389/fpubh.2021.650022)

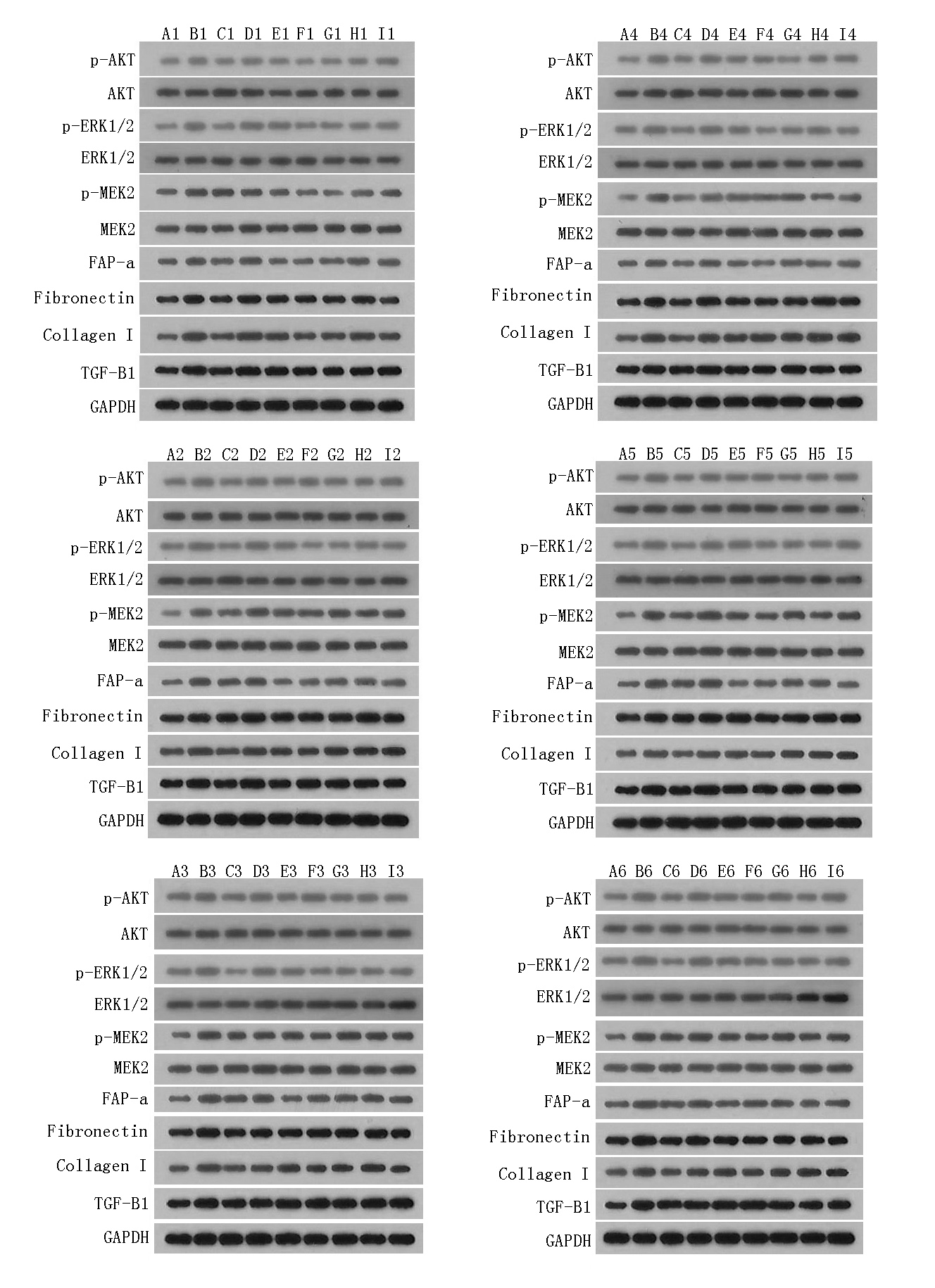

Supplement: Supplementary file 1 [file Image_1.JPEG]

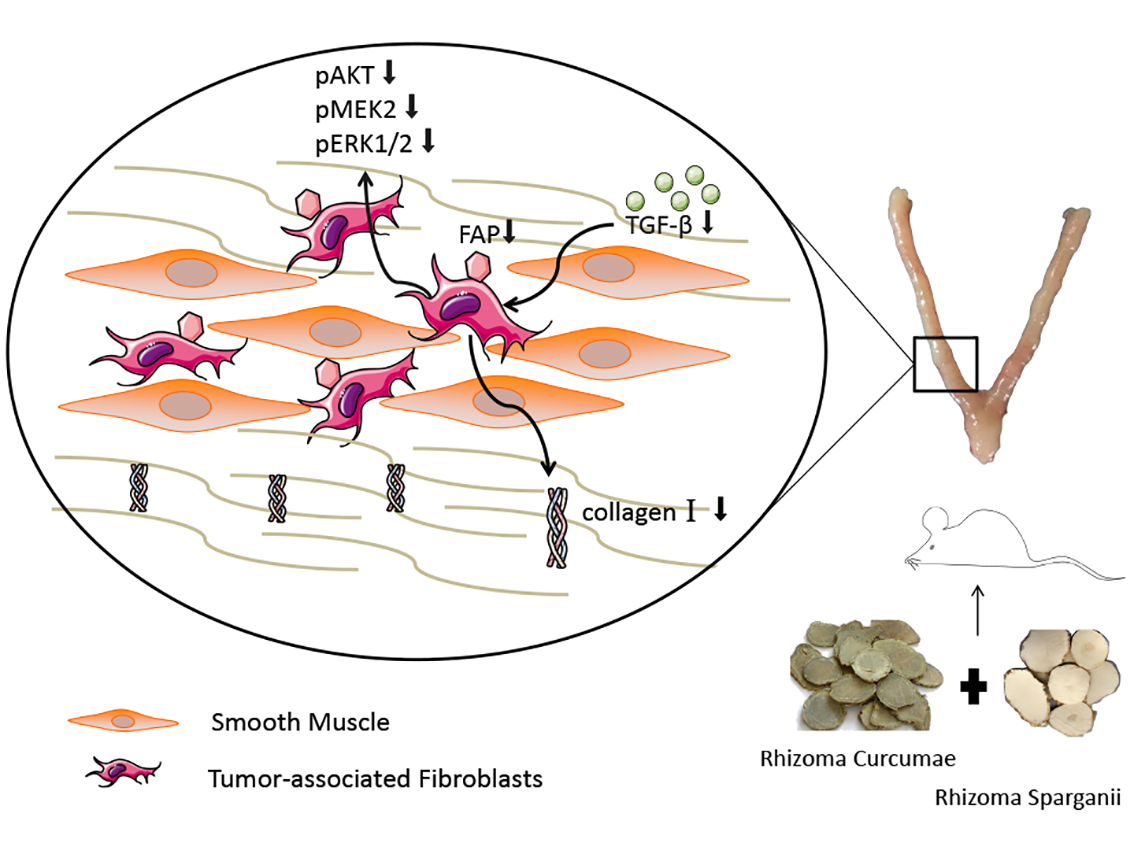

Supplement: Supplementary file 2 [file Image_2.JPEG]
